# Environmental radiation alters the gut microbiome of the bank vole *Myodes glareolus*

**DOI:** 10.1038/s41396-018-0214-x

**Published:** 2018-07-09

**Authors:** Anton Lavrinienko, Tapio Mappes, Eugene Tukalenko, Timothy A. Mousseau, Anders P. Møller, Rob Knight, James T. Morton, Luke R. Thompson, Phillip C. Watts

**Affiliations:** 1 Department of Ecology and Genetics, University of Oulu, 90014, Oulu Finland; 2 Department of Biological and Environmental Science, University of Jyväskylä, 40014 Jyväskylä, Finland; 3 Institute of Biology and Medicine, Taras Shevchenko National University of Kyiv, Kyiv, 03022 Ukraine; 4 Department of Biological Sciences, University of South Carolina, Columbia, SC 29208 USA; 5 Ecologie Systématique Evolution, Université Paris-Sud, CNRS, AgroParisTech, Université Paris-Saclay, 91405 Orsay Cedex, France; 6 Department of Pediatrics, University of California San Diego, La Jolla, CA 92037 USA; 7 Department of Computer Science and Engineering, University of California San Diego, La Jolla, CA 92037 USA; 8 Center for Microbiome Innovation, University of California San Diego, La Jolla, CA 92037 USA; 9 Department of Biological Sciences and Northern Gulf Institute, University of Southern Mississippi, Hattiesburg, MS USA; 10 Atlantic Oceanographic and Meteorological Laboratory, National Oceanic and Atmospheric Administration, stationed at Southwest Fisheries Science Center, National Marine Fisheries Service, La Jolla, CA USA

## Abstract

Gut microbiota composition depends on many factors, although the impact of environmental pollution is largely unknown. We used amplicon sequencing of bacterial 16S rRNA genes to quantify whether anthropogenic radionuclides at Chernobyl (Ukraine) impact the gut microbiome of the bank vole *Myodes glareolus*. Exposure to elevated levels of environmental radionuclides had no detectable effect on the gut community richness but was associated with an almost two-fold increase in the Firmicutes:Bacteroidetes ratio. Animals inhabiting uncontaminated areas had remarkably similar gut communities irrespective of their proximity to the nuclear power plant. Hence, samples could be classified to high-radiation or low-radiation sites based solely on microbial community with >90% accuracy. Radiation-associated bacteria had distinct inferred functional profiles, including pathways involved in degradation, assimilation and transport of carbohydrates, xenobiotics biodegradation, and DNA repair. Our results suggest that exposure to environmental radionuclides significantly alters vertebrate gut microbiota.

## Introduction

Our understanding of the factors that shape gut microbiome is dominated by studies of humans [1] or laboratory animals [2]. In contrast, the processes that determine the composition of gut microbiomes in wildlife are poorly known but include, for example, host diet (in wild mice, [3]) and habitat quality (in primates, [4]). Whether persistent pollutants impact the gut microbiomes of wildlife in their natural habitat is not known, although exposure to various chemicals, heavy metals [5], or ionizing radiation [6] can change the gut microbiota composition of laboratory rodents.

Pollution by radionuclides is a potential source of genotoxicity to humans and wildlife [7]. Animals inhabiting the Chernobyl Exclusion Zone (CEZ) provide the best-studied model of the biological impact of exposure to environmental radionuclides [8]. Detrimental effects of a chronic exposure to radiation on wildlife include elevated DNA damage [7] and upregulation of DNA repair genes [9], increased oxidative stress [10], and an increase in mutation rate [11]. Any yet, little is known about the effects of environmental radiation on microorganisms. While some bacteria from the CEZ have a capacity for adaptation to environmental radioactivity [12, 13], other studies reported a decline in diversity with increased radiation exposure [14]. However, no study has quantified the impact of exposure to environmental radiation on host-associated gut microbial communities in wild animals.

## Methods and results

**Fig. 1:**
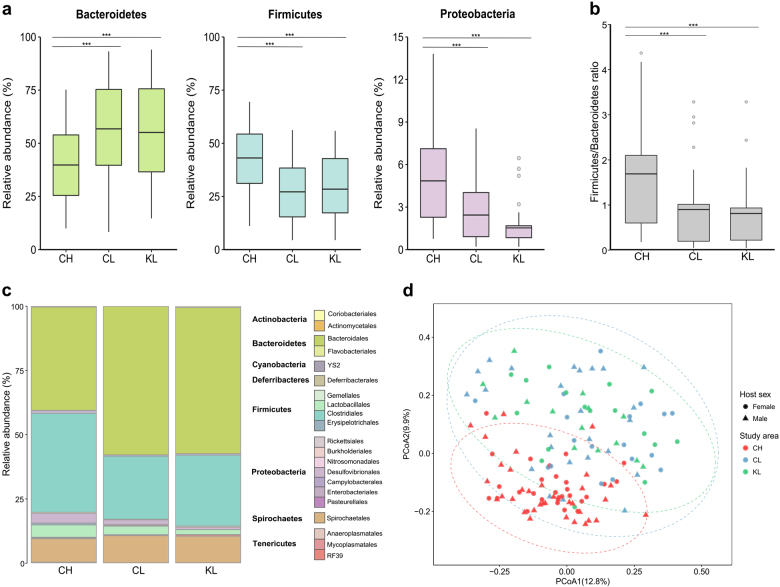
Radiation-associated differences in bank vole gut microbiota community composition and beta diversity. **a** Relative abundances of three major bacterial phyla and **b** Firmicutes to Bacteroidetes ratio (F:B) in the gut microbiota of bank voles inhabiting areas that differ in levels of environmental radiation. Asterisks indicate significant differences among areas contaminated (CH) and uncontaminated (CL) with radionuclides within the Chernobyl Exclusion Zone and an uncontaminated area near Kyiv (KL), Ukraine (Bonferroni-corrected Kruskal–Wallis test). ***P < 0.001. **c** Mean relative abundance of bacterial taxa at order level in the bank vole gut microbiota. Unassigned taxa (<2.3%) are not shown. **d** PCoA on Bray–Curtis dissimilarity distances between bank vole gut microbiota profiles among the three study areas. Each point represents a single sample, shape indicates host sex, colored according to study area: CH, red (*n* = 63); CL, blue (*n* = 43); KL, green (*n* = 31). Ellipses represent a 95% CI around the cluster centroid. Clustering significance by treatment group was determined by adonis, *P* < 0.001

To quantify whether exposure to environmental radiation impacts the gut microbial community of a wild rodent, the bank vole *Myodes glareolus*, we sequenced the V4 region of the 16S rRNA genes from the gut microbiota of 137 bank voles (Supplementary Table 1). Bank voles were sampled from three study areas that differed in the levels of environmental radiation, and represented two treatments: (1) high (CH *n* = 63, mean = 30.1 µSv/h) and (2) low (CL *n* = 43, mean = 0.25 µSv/h and KL *n* = 31, mean = 0.33 µSv/h) radiation contamination (Supplementary Figure 1, see detailed descriptions of the methods and results in the Supplementary Information). Neither community richness nor evenness differed significantly (*P* > 0.05) between samples grouped by study area (all pairwise comparisons of CH, CL, and KL) or were affected by host sex (Supplementary Figure 2, Supplementary Table 2). Significant (*P* = 0.001) differences in beta diversity (Fig. 1d, Supplementary Figure 3) were observed between CH and both CL and KL, but not due to effects of sex, a contamination by sex interaction, or due to variation in bank vole body mass, head width (a proxy for age), or gravidity status (for females) (Supplementary Table 3).

Radiation, but not individual level predictors such as sex, body mass, or head width (a proxy for age), was identified as a significant predictor of the abundance of Bacteroidetes (*P* = 0.0001), Firmicutes (*P* < 0.0001), and Proteobacteria (*P* = 0.001) (Supplementary Table 4). Notably, we found an almost two-fold, significant (*P* < 0.001) decrease in the ratio of Firmicutes to Bacteroidetes (F:B) sequences from 1.7 in the radioactively contaminated CH area to <0.9 in the uncontaminated areas CL (F:B = 0.87) and KL (F:B = 0.78) (Fig. 1a, b, Supplementary Tables 5 and 6). This pattern was apparent across replicate sites (Supplementary Figure 4) and taxonomic levels (Fig. 1c, Supplementary Table 6) in each treatment, indicating that the F:B ratio in bank vole guts is associated with the level of environmental radiation.

**Fig. 2:**
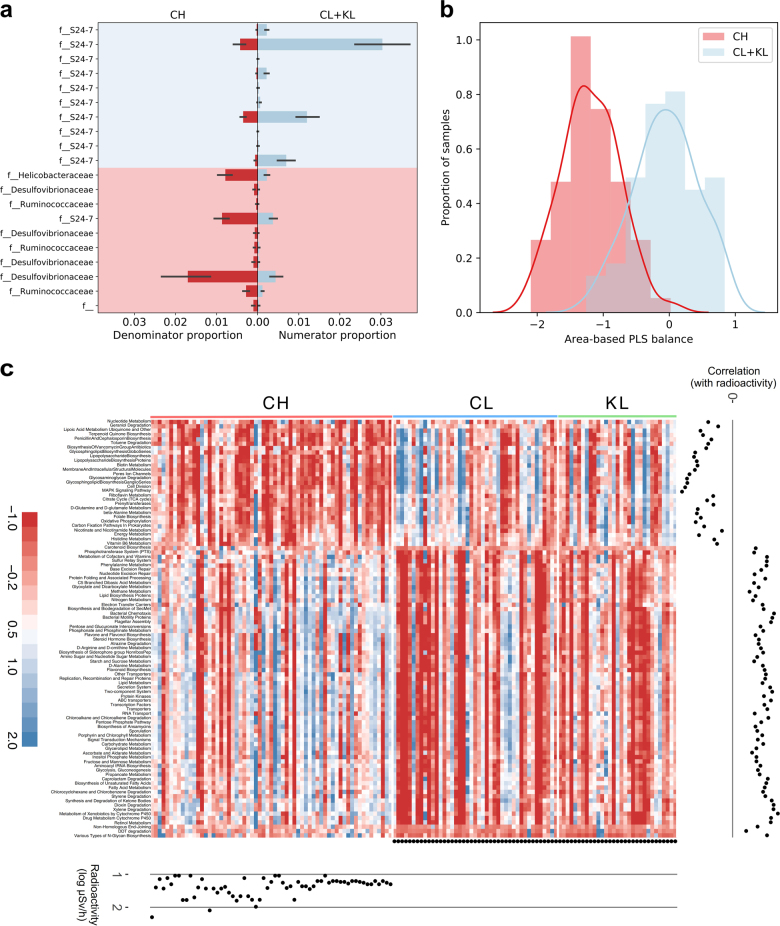
Balance Trees analysis and PICRUSt functional predictions for the bank vole gut microbiota. Partial least squares (PLS) balance analysis of **a** area-differentiated OTUs (shown are the proportions in the two groups of the top 10 OTUs in each group based on PLS score) and **b** classification of samples to study areas based on OTU composition. **c** Heat map of the KEGG level-3 functional pathways that significantly differed between contaminated (CH) and both uncontaminated areas (CL and KL). Pathway counts were normalized as implemented in superheat v0.1.0 R package, abundance of each pathway is indicated by a gradient of color from red (low abundance) to blue (high abundance). Full lists of pathways associated with radiation exposure can be found in Supplementary Table 11

These trends were also verified beyond the phylum level in independent predictions by using a combination of partial least squares (PLS) regression and balances [15] (Supplementary Information). The area-based balance could differentiate between the CH and the CL/KL areas (AUC = 0.957, *F*-statistic = 204.7, *P* = 9.18 × 10^−29^), with a mean cross-validation prediction accuracy of 0.917 (Fig. 2a, b, Supplementary Tables 7 and 8). This pattern was robust, even when the analysis was limited to 10 samples per study area (Supplementary Table 8). Using the radiation-based balance, we could predict radiation levels (Pearson’s *r* = –0.766, *P* = 1.46 × 10^−27^) (Supplementary Figure 5, Supplementary Tables 9 and 10, see Supplementary Information for results).

We used PICRUSt [16] to make functional predictions of bank vole gut microbiota. Analyses using both PLS and Kruskal–Wallis identified core bacterial functions strongly associated with the study area (CH vs. CL/KL) (Fig. 2c, Supplementary Table 11). The striking similarity in the functional profiles of the uncontaminated areas, despite their spatial separation (Supplementary Figure 1) and potential habitat differences, and the contrast between the uncontaminated and contaminated areas reinforces the relevance of radioactivity on gut microbial community function.

## Discussion

Exposure to anthropogenic radionuclide contamination presents diverse risks to living organisms [7], but its effects on gut microbiota are unknown. Our analysis of the gut microbiota of a wild vertebrate exposed to environmental radiation uncover major changes to the gut microbiome composition and inferred functional profiles.

Extensive inter-individual variation in gut microbiota profiles (Fig. 1d) is a typical feature of host heterogeneity in factors such as age, genotype [17], sex, and diet [3]. While the use of diverse sampling locations will likely add noise, it is an essential part of our experimental design that the treatment (radiation) effect is observed outside one specific location such as the Red Forest (one of the most contaminated sites in the CEZ) [18]. No other potential predictors (e.g. body mass, age, sex) of gut bacterial abundance were found, in contrast to the general effect of radiation exposure (Supplementary Tables 3 and 4).

The CEZ presents a mosaic of radionuclides deposition, where contaminated and uncontaminated areas can be separated by ~1.5 km [19]. Bank voles may move up to 1 km within a breeding season [20], and thus individuals from uncontaminated areas within the CEZ (CL), but not individuals in the area around Kyiv (KL), have the opportunity to be exposed to radionuclides prior to capture [9]; with this in mind, the differences in gut microbiota composition between CH and CL, and the similarity between CL and KL, are striking. The implication is that the gut microbial community responds rapidly and at a small scale to host habitat, consistent with laboratory experiments that show how external stimuli can drive changes in gut microbiome within a few days [5, 21].

Changes in the gut community composition might reflect bacterial radiosensitivity. Interestingly, some members of the *Desulfovibrionaceae* can tolerate high radiation levels (CH) and have a potential for bioremediation of radionuclides [22], raising the possibility that it has a detoxifying role in the bank vole gut. It is not known whether the comparatively low and chronic dose of radionuclides (~2–10 mGy/d) that wildlife experience within the CEZ [23] could impose differential mortality within the gut bacterial community. However, enrichment of DNA repair pathways (Fig. 2c, Supplementary Table 11) in contaminated areas implies an increased effort to repair bacterial DNA associated with elevated levels of environmental radiation.

Selection for a more distinct gut microbiota in radioactively contaminated areas primarily reflects a replacement of Bacteroidetes by Firmicutes. In a broad context, the F:B ratio within the gut is affected by diet [2, 17] and is associated with bacterial metabolic potential [24]. Bank voles have a catholic diet that includes seeds, leaves, roots, grasses, fruit and fungi and, occasionally, insects, and other invertebrates [25]. Gut communities dominated by Bacteroidetes can exploit various dietary substrates [26] and thus, could allow efficient use of diverse foods, indicating the likely adaptive state in nature. Firmicutes tend to metabolize otherwise indigestible resistant starch (Clostridiales, Lactobacillales) and plant cell wall glycans (*Ruminococcaceae*, *Lachnospiraceae*) [26]. Bank voles inhabiting radioactively contaminated areas (CH) thus exhibit an apparent dietary shift, which is also supported by the inferred metabolic functions (Fig. 2c, Supplementary Table 11). There are no detailed biodiversity surveys of the CEZ and surrounding areas, but a change in diet associated with environmental radioactivity might reflect the reduced abundance of arthropods in the more contaminated areas within the CEZ [27]. Alternatively, bank voles inhabiting CH areas might actively increase the amount of plant-based foods to produce more of the end products of fermentation of dietary fiber (short-chain fatty acids, SCFAs) [28].

SCFAs can confer additional benefits to the host beyond energy harvest by acting as physiological and immune regulators [26, 29]. Gut bacteria that produce butyrate may protect against genotoxins as this SCFA affects DNA repair systems and antioxidant levels [30] and can reduce oxidative stress that is widely associated with animals inhabiting contaminated areas within the CEZ [10]. Butyrate is produced by many Firmicutes, specifically members of the Clostridiales, *Ruminococcaceae* and *Lachnospiraceae* [31], that are significantly more abundant in CH, but is rarely associated with the SCFA profiles of Bacteroidetes [26]. Enrichment of Firmicutes (Fig. 1a, b, Supplementary Table 6) thus is a plausible method of mitigating effects of elevated oxidative stress concomitant with inhabiting an area contaminated by radionuclides. This hypothesis warrants further studies that could quantify SCFAs profiles and thoroughly assess host diet.

In conclusion, bank voles exposed to anthropogenic environmental radiation experience substantial changes in the composition of their gut microbiota and major changes in bacterial function. Gut microbial composition can facilitate adaptation [32], and might be an important component of mounting an effective response to conditions within the CEZ. The positive and negative adaptive and health consequences of changes to wildlife gut microbiota remain to be quantified.

## Electronic supplementary material

Supplementary Information

Supplementary Table 1

Supplementary Table 2

Supplementary Table 3

Supplementary Table 4

Supplementary Table 5

Supplementary Table 6

Supplementary Table 7

Supplementary Table 8

Supplementary Table 9

Supplementary Table 10

Supplementary Table 11
