# Supplementary material for: Environmental radiation alters the gut microbiome of the bank vole Myodes glareolus
Source: ISME J. 2018 Jul 9;12(11):2801–6. doi: 10.1038/s41396-018-0214-x (PMC6193954; doi:10.1038/s41396-018-0214-x)
Supplement: Supplementary file 1 — Supplementary Information [file 41396_2018_214_MOESM1_ESM.docx]

**Supplementary Information**

Environmental radiation alters the gut microbiome of the bank vole *Myodes glareolus*

Anton Lavrinienko^1^*, Tapio Mappes^2^, Eugene Tukalenko^2,3^, Timothy A. Mousseau^4^, Anders P. Møller^5^, Rob Knight^6,7,8^, James T. Morton^6,7^, Luke R. Thompson^9,10^, Phillip C. Watts^1^

^1^Department of Ecology and Genetics, University of Oulu, 90014, Finland

^2^Department of Biological and Environmental Science, University of Jyväskylä, 40014, Finland

^3^Institute of Biology and Medicine, Taras Shevchenko National University of Kyiv, 03022, Ukraine

^4^Department of Biological Sciences, University of South Carolina, Columbia, SC 29208, USA

^5^Ecologie Systématique Evolution, Université Paris-Sud, CNRS, AgroParisTech, Université Paris-Saclay, 91405 Orsay Cedex, France

^6^Department of Pediatrics, University of California San Diego, La Jolla, CA 92037, USA

^7^Department of Computer Science and Engineering, University of California San Diego, La Jolla, CA 92037, USA

^8^Center for Microbiome Innovation, University of California San Diego, La Jolla, CA 92037, USA

^9^Department of Biological Sciences and Northern Gulf Institute, University of Southern Mississippi, Hattiesburg, MS, USA

^10^Atlantic Oceanographic and Meteorological Laboratory, National Oceanic and Atmospheric Administration, stationed at Southwest Fisheries Science Center, National Marine Fisheries Service, La Jolla, CA, USA

*Corresponding author: Anton Lavrinienko.

Email: [Anton.Lavrinienko@oulu.fi](mailto:Anton.Lavrinienko@oulu.fi)**Supplementary Materials and Methods**

*Sample sites*

Bank voles were caught at 62 trapping locations within northern Ukraine (Supplementary Table 1), during May-June 2016. Soil radiation levels at all trapping locations were measured at 1 cm above the ground using a Geiger-Mueller dosimeter (Inspector, International Medcom Inc., CA, USA) calibrated to Sieverts (Sv). The ambient radiation dose rate measurements that derive from the energies emitted by radionuclides present in a study area are repeatable among days and even years (Møller and Mousseau, 2013). The CEZ presents a mosaic of radiation contamination (Chesser *et al.*, 2004), allowing bank voles to be caught from areas where the soil is contaminated by radionuclides, and apparently uncontaminated areas where soil radiation levels are not significantly elevated above background level.

Our trapping locations represent three general study areas (Supplementary Figure 1) that contained multiple (2-3) replicate sites within each area, separated by 9-33 km, which is beyond bank vole dispersal abilities (up to 1 km) (Kozakiewicz *et al.*, 2007). Briefly, (1) Chernobyl High (CH) representing three separate replicates within the CEZ, with elevated soil radiation levels; (2) Chernobyl Low (CL) that consisted of two replicates within the CEZ, where radiation levels are not significantly elevated above background; and (3) Kyiv Low (KL) representing two replicates that are near Kyiv and thus outside the CEZ (Supplementary Figure 1): elevated levels of soils radiation have not been detected at this area. Note, CH sites contained significantly (Bonferroni-corrected Kruskal–Wallis test, *P*<0.0001) higher levels of radionuclides (hereafter, contaminated area; mean=30.1; range, 10–198.7 µSv/h; n=63 individuals) compared with (2) CL (mean=0.25; range 0.1–0.6 µSv/h; n=43) and (3) KL (mean=0.33; range 0.2–0.6 µSv/h; n=31), with the latter two sites both lacking elevated levels of environmental radiation (hereafter, uncontaminated areas) and not differing (*P*>0.05) in level of environmental radioactivity among each other (Supplemental Table 1). Hence, the three study areas were designed to contrast in levels of environmental radiation and represent two treatments with high (CH) and low (CL and KL) radiation contamination.

*Bank vole trapping and faecal sample collection*

Bank voles were caught using Ugglan Special2 multiple-capture live traps (Grahnab, Sweden) (Lehmann *et al.*, 2016). At each location, 16 traps were placed in a 4x4 grid, with an inter-trap distance of 20 m. The trapping period was up to three consecutive nights in each location. Traps were initially set in the late afternoon and animals were collected early each following morning. At time of arrival to the field laboratory at Chernobyl, captured animals were placed in individual Makrolon Type III cages (43x26x15 cm) that had been ethanol-sterilized. Animals were monitored for 2 hours and immediately after defecation, faecal pellets were frozen at -20^○^C, and stored at -80^○^C prior to DNA extraction (within 3 months). Upon faeces collection and when other non-destructive handling procedures were completed, most of the animals were released back to the free-range habitat, juvenile individuals were euthanized by cervical dislocation to sample tissues for the purposes of other studies. Sampling was completed within three weeks to minimize potential effects of seasonal shift in gut microbial community (Maurice *et al.*, 2015). To avoid potential batch effects and systematic bias, samples from each treatment group were processed (*e.g*. storage, transportation, DNA isolation and sequencing) at random. All procedures were performed in accordance with international guidelines and regulations for the use of animals in research. The study was approved by the Finnish Ethical Committee (ESAVI/7256/04.10.07/2014).

Bank vole length (nose to anus) and head width was measured to the nearest 0.1 mm with a digital caliper, and weight was measured to the nearest 0.1 g (Mettler Toledo XS204). Three age classes were allocated according to head width (Kallio *et al.*, 2014): juveniles (~1 month, 11.6–12 mm) and two age categories of adults (~2-5 months, 12.1–13.4 mm and >10 months, 13.5–14 mm) (Supplementary Table 1). Visual assessment of sex and maturity at capture was performed relative to animal phenology (*i.e.* descended testes for males, gravid, lactation or with a perforate vagina for females).

*16S rRNA gene sequencing*

Total DNA was extracted from 0.1 g of faecal material (n=137 individuals, with one sample per animal) using a PowerFecal DNA Isolation kit (MoBio Laboratories, Carlsbad, CA, USA) following the manufacturer’s instructions. DNA concentration was quantified with a Qubit 2.0 fluorometer (Invitrogen, Carlsbad, CA, USA).

PCR amplification of the 16S ribosomal RNA (rRNA) gene and library preparation were performed following to the Earth Microbiome Project protocol, ([www.earthmicrobiome.org/protocols-and-standards](http://www.earthmicrobiome.org/protocols-and-standards)). Briefly, the V4 variable region (~254 bp) of the 16S rRNA gene was amplified using the original 515F/806R primer pair (Caporaso *et al.*, 2012). PCR products were purified with an AMPure XP PCR Purification kit (Agencourt, Danvers, MA, USA). The final library was quantified using an Agilent DNA 1000 kit on an Agilent 2100 Bioanalyzer (Agilent technologies, Waldbronn, Germany), and by real-time quantitative PCR EvaGreen (Biotium, Hayward, CA, USA). Barcoded amplicons were sequenced on an Illumina MiSeq to provide paired-end 250-bp reads by Beijing Genomics Institute, Hong Kong, China ([www.bgi.com/global/](http://www.bgi.com/global/)).

*Read data processing*

Read data were trimmed according to quality (Q30 base threshold) and de-multiplexed at the sequencing facility. Overlapping paired-end reads were assembled using PEAR v.0.9.10 (Zhang *et al.*, 2014), with specified minimum (-n 252) and maximum (-m 254) length of assembled sequences. After quality control, there was a total of 7 235 678 reads that represented an average of 52 815 reads per sample (range=22 402-130 342).

Data were processed using QIIME v.1.9.1 (Caporaso *et al.*, 2010). Potential chimeric sequences were identified and removed using UCHIME (Edgar *et al.*, 2011), with the retained (non-chimeric) sequences clustered into operational taxonomic units (OTUs) using the combination of SortMeRna and SUMACLUST methods implemented by the open-reference OTU-picking pipeline within QIIME. Bacterial taxonomy was assigned at 97% sequence similarity against the GreenGenes v.13_8 database (McDonald *et al.*, 2012). Low-abundance (<0.005% of read data) OTUs were removed (Bokulich *et al.*, 2013). OTU selection yielded an average of 43 054 reads/sample (range=18 113-105 937) (Supplementary Table 1) that represented 1 312 OTUs over all samples (average of 704 OTUs/sample, range=387-929 OTUs/sample) (Supplementary Table 1). OTU data were rarefied to 18 000 reads/sample to avoid biases caused by variation in sequencing depth among samples (Weiss *et al.*, 2017).

An assessment of functional composition associated with the gut microbiota was made using PICRUSt v.1.1.0 (Langille *et al.*, 2013). Low abundance OTUs and OTUs without a match in the GreenGenes v.13_8 database were removed and these filtered data were rarefied to a depth of 10 000 reads/sample. Predicted metagenomes were classified according to KEGG orthologs (KOs) and summarized using KEGG pathways (Level-2 and 3). A relatively high (mean=0.21, SD=0.05) weighted Nearest Sequenced Taxon Index (NSTI, average branch length separating our OTUs and the available reference bacterial genomes) in our data is comparable with use of PICRUSt in other non-model mammals (Langille *et al.*, 2013; Zeng *et al.*, 2015).

Sequence data are available at the European Bioinformatics Institute (EBI) database (via Qiita, <https://qiita.ucsd.edu/>, study ID 11360) with EBI accession number ERP104266. All other data are available upon request from the authors.

*Statistical analyses*

Statistical analyses were performed using R v.3.3.3 unless otherwise stated. Estimates of alpha diversity (richness - number of observed OTUs, evenness - Shannon index) were calculated using phyloseq R package v.1.19.1 (McMurdie and Holmes, 2013). Potential differences in alpha diversity among the three study areas were quantified using Kruskal–Wallis tests, followed by a Dunn’s *post hoc* test using the dunn.test package (Dinno, 2016) for R and a Bonferroni correction for multiple testing.

To quantify differences in gut microbial community composition among the three areas (CH, CL and KL), we performed Kruskal–Wallis tests (followed by a Bonferroni correction for multiple testing) across bacteria taxonomic hierarchy. We also identified potential predictors of bacterial phyla abundance using generalized linear modeling (GLM). Bank vole body mass, head width (a proxy for age) (Kallio *et al.*, 2014), sex (male, female), radiation exposure (high: CH, low: CL and KL) and, for females, gravid status (pregnant, not pregnant) were used as potential explanatory variables for abundance of gut bacterial phyla. The full model used body mass and head width as continuous variables, other predictors as factors and allowed an interaction between sex and radiation exposure. Model selection was based on Akaike Information Criterion (AIC) adjusted for sample size using AICc for model ranking (Bolker *et al.*, 2009). The most parsimonious model within 2 AICc units from the model with the lowest AICc was considered to be the best model supported by the data and subsequently used for statistical inference. Model selection was carried out using the dredge function in MuMIn v.1.15.6 (Barton, 2016).

We used a combination of partial least squares (PLS) regression and balances (Morton *et al.*, 2017) to detect differentially represented taxa among treatments (*i.e*. to identify OTUs strongly associated with radiation level in addition to study area). PLS can identify the features (OTUs) that best distinguish treatment groups. Balances are useful for accounting for interpretation issues associated with compositionality in 16S rRNA amplicon data (Morton *et al.*, 2017) by performing inferences on log-ratios of sub-communities rather than on proportions. The PLS approach serves as a classifier for OTUs by assigning OTUs into one of three classes: (1) associated with the first sample category, (2) associated with the second sample category, or (3) associated with neither category.

To estimate the balances to build the classifier, PLS was first performed on a log-transformed table, where the means of the rows and the columns were centered around zero. A pseudocount was added to avoid taking the logarithm of zero. Only the first component from the PLS analysis was considered, and the resulting loadings from the OTU table were analyzed to create the balance. The resulting loadings matrix assigns a score for how strongly associated a given feature was to a single environment. Highly negative scores are strongly associated with one environment, highly positive scores are strongly associated with the other environment, and scores close to zero are suspected to be weakly associated with either environment. From simulations, it has been shown that OTUs that are weakly associated with both environments follow a Gaussian distribution around zero (<https://github.com/knightlab-analyses/pls-balances>). Using the Gaussian distribution as a null distribution for the weakly associated OTUs, we fitted Gaussian mixture models. We then used the intersections between the fitted Gaussians to assign hard thresholds for assigning the OTUs to one of the two sample environments or neither environment. Here we created two PLS models, one building a balance to classify samples and OTUs with respect to the sampling area (*i.e.* CH vs CL/KL), and another balance to predict the radiation levels. Four-fold cross validation was used to evaluate prediction accuracy and ensure that overfitting is not occurring.

Beta-diversity was estimated using the Bray-Curtis dissimilarities and weighted UniFrac distances between samples, as calculated by phyloseq v.1.19.1 (McMurdie and Holmes, 2013). Both Bray-Curtis and weighted UniFrac metrics consider the differences in taxon abundance, while UniFrac also incorporates phylogenetic distances between bacterial phylotypes (Lozupone *et al.*, 2011). Differences among samples were visualized by Principal Coordinates Analysis (PCoA), with statistical significance calculated using the permutation MANOVA (PERMANOVA) (999 permutations) implemented by the adonis function in vegan v.2.4-2 (Oksanen *et al.*, 2016). To assess the stability of the PCoA clusters and examine any potential effect of sequencing depth, we performed the jackknife resampling procedure implemented in QIIME. We repeatedly (10 times) resampled 10% of the sequence data from each sample (1800 reads/sample) and aggregated the results as confidence ellipsoids (inter-quartile range) around the sample average.

Statistical comparisons of bacterial functional predictions were made for level-2 and level-3 KEGG pathways using (1) Kruskal–Wallis tests followed by a Benjamini-Hochberg False Discovery Rate (FDR) correction (Benjamini and Hochberg, 1995) and (2) a PLS analysis that classified inferred KEGG pathways to the study areas (*i.e.* CH vs CL/KL) using the pathway tables (instead of the OTU table as described above). The KEGG level-3 pathways that significantly differed between contaminated and both uncontaminated areas (excluding predictions related to Human Diseases and Organismal Systems categories) were plotted as a heat map using superheat v.0.1.0 R package (Barter, 2017).

**Supplementary Results**

We sampled the gut microbiota from 137 bank voles (73 males and 64 females) from areas within and outside the Chernobyl Exclusion Zone (CEZ) (Supplementary Figure 1) that contrasted in their level of environmental radiation. Bank vole gut microbiota is composed of eight bacterial phyla, but dominated by the Bacteroidetes (48%) and Firmicutes (35%). The six bacterial phyla detected at comparatively low levels were: Spirochaetes (10%), Proteobacteria (4%), Tenericutes (<0.1%), Actinobacteria (<0.1%), Cyanobacteria (<0.1%) and Deferribacteres (<0.1%) (Figure 1c, Supplementary Table 5). This community composition is consistent with gut microbial communities in other wild rodents (Maurice *et al.*, 2015). Bacteroidetes were dominated (>96%) by an unclassified genus from the *S24-7* family (Supplementary Table 5). In contrast, the Firmicutes were more diverse, with representative OTUs from *Clostridiaceae*, *Ruminococcaceae*, *Lachnospiraceae* and *Lactobacillaceae* families, albeit with unclassified taxa from the Clostridiales order dominating (>46%) the Firmicutes. From multiple taxa assigned to Proteobacteria, genus *Desulfovibrio* from the *Desulfovibrionaceae* family was dominant (>62%) (Supplementary Table 5).

*Radiation-associated differences in bank vole gut microbiota community composition*

Importance of radioactivity on the abundance of Firmicutes and Bacteroidetes was evident across taxonomic levels (Figure 1c, Supplementary Table 6). For example, within the Bacteroidetes the abundance of bacteria from unclassified genera within the *S24-7* family was significantly (*P*<0.005) lower in samples from CH than CL and KL. Abundances of bacteria from ‘unclassified Clostridiales’ and *Ruminococcacae* (*~*66% of the Firmicutes) were significantly (*P*<0.05) higher in CH than in CL and KL. In addition, *Lachnospiraceae* and *Lactobacillaceae*, other family groups of Firmicutes, were significantly (*P*<0.001) more abundant at CH than in CL or KL respectively (Supplementary Table 6). Within Proteobacteria, taxa from the *Flexispira* genus were significantly (*P*<0.0001) more abundant in samples from CH than CL and KL. Abundance of the *Desulfovibrio* genus varies among areas, but was more common in CH (Supplementary Table 5).

Partial least squares (PLS) analysis identified OTUs strongly associated with study area (CH or CL/KL) and radiation level. The area-based balance was able to clearly differentiate between the CH and the CL/KL areas (AUC=0.957, *F*-statistic=204.7, *P*=9.18x10^-29^), with a mean cross-validation prediction accuracy of 0.917. The area-based PLS balance comprised 281 OTUs associated with the CL/KL areas with a high representation of taxa from family *S24-7* (Bacteroidetes) and 166 OTUs associated with the CH area with a high representation of taxa from Firmicutes phylum (Clostridiales order, *Ruminococcaceae, Lachnospiraceae, Desulfovibrionaceae* families) (Figure 2a, Supplementary Table 7 and Supplementary Table 8).

To quantify the robustness of these data (*i.e*. the extent to which we can detect an effect of environmental radiation depends on sample size), we repeated the PLS regression analysis 10 times, but taking a random subsample (individuals) per each study area. For five of these subsamples, we resampled n=30 individuals (with replacement) per study area, and for the other 5 subsamples we resampled n=10 individuals (with replacement) per study area. With random sampling of n=30 samples per study area (about half of the original sample size for CH), we could differentiate between the CH and CL/KL areas (range of 5 subsamplings: AUC=0.968–0.995, F-statistic=124.5–178.6, P=6.82x10^–23^–1.57x10^–18^). With just 10 samples per study area, we still could differentiate between the CH and CL/KL areas (range of 5 subsamplings: AUC=0.995–1.0, F-statistic=73.8–277.8, P=4.55x10^–16^–2.45x10^–9^) (Supplementary Table 8).

Using the radiation-based balance, we were able to predict radiation levels (Pearson’s *r*=–0.766, *P*=1.46x10^–27^). Similar to the area-based balance analysis, 281 OTUs were associated with low radiation within the numerator of the radiation-based balance, of which 208 were estimated to originate from the *S24-7* family (Bacteroidetes) (Supplementary Figure 5, Supplementary Table 9 and Supplementary Table 10). Of the 175 OTUs associated with high radiation, there was high representation of taxa from the order Clostridiales. Furthermore, 42 of these taxa came from the *Ruminococcaceae* family, 20 from the *Lachnospiraceae* family and 20 OTUs from the *Desulfovibrionaceae* family.

*Variation in bank vole gut microbial alpha and beta diversity*

Alpha diversity (number of OTUs, Shannon Index) did not differ significantly (*P*>0.05) between samples from contaminated and uncontaminated areas regardless of host sex (Supplementary Figure 2, Supplementary Table 2). Differences in the gut microbiome associated with their bank vole host being exposed to elevated environmental radiation thus consists of replacement of key OTUs (the change in abundances described above) rather than major differences in the presence or absence of specific bacterial taxa.

Bank voles exhibit considerable inter-individual variation in their gut microbiota communities. While there is substantial overlap in the gut microbial communities among treatments, the uncontaminated areas are characterized by larger inter-individual variation than samples from the contaminated CH areas. The cluster of samples from CH was separated from CL and KL samples principally on the second PCoA axis for both Bray-Curtis (Figure 1d) and weighted UniFrac metrics (Supplementary Figure 3). These sample groups remained even when the PCoA analysis was limited to 10% of the available sequencing data per each sample (Supplementary Figure 6). Irrespective of the distance metric used, significant (*P*=0.001) differences in beta diversity were observed between the contaminated and uncontaminated areas, but not between sexes, contamination by sex interaction or due to the variation in bank vole body mass, head width (a proxy for age) and gravidity status (for females) (Supplementary Table 3). Despite substantial inter-individual variation, consistent with the deliberately heterogeneous sampling, our data illustrate how inhabiting an area with elevated levels of radionuclide contamination impacts gut microbiome profiles.

*Functional predictions for bank vole gut microbiota communities*

The functional predictions of bank vole gut microbiota from both treatments shared a common set of KEGG pathways comparable to that of other herbivorous and omnivorous mammals (Koropatkin *et al.*, 2012) (Supplementary Table 11). No statistical differences in KEGG level-2 pathway categories were apparent between the uncontaminated areas CL and KL, indicating a stable set of core functions and remarkably similar metabolic potential, consistent with their similar gut microbiota communities (Figure 1c, Supplementary Table 6). By contrast, 17 (out of 39) KEGG level-2 pathways differed significantly (*P*<0.05) between contaminated and both uncontaminated areas (Supplementary Table 11); an additional eight KEGG level-2 pathways differed significantly between CH and CL, but these are not considered to present a major effect of radiation exposure as the effects are limited to the CEZ. KEGG level-2 pathways that were overrepresented in samples from CH included those associated with cellular processes (cell growth and death, cell motility), environmental information processing (membrane transport and signal transduction), genetic information processing (transcription) and metabolism (carbohydrate metabolism, xenobiotics biodegradation and metabolism). KEGG level-2 pathways that were overrepresented in the uncontaminated CL and KL areas were metabolism (glycan biosynthesis and metabolism). A similar pattern is observed at KEGG level-3 pathways, where just three (out of 216) significant differences were observed between CL and KL, while 116 pathways differed between contaminated and both uncontaminated areas (Figure 2c, see Supplementary Table 11 for full details of pathways).

PLS analysis identified KEGG pathways strongly associated with the study area (*i.e.*, CH vs CL/KL). The area-based balance was able to differentiate level-2 (AUC=0.749, *F*-statistic=33.12, *P*-value=5.63x10^-8^) and level-3 (AUC=0.760, *F*-statistic=34.06, *P*-value=3.82x10^-8^) pathways between the CH and the CL/KL areas, with results fully consistent with the Kruskal-Wallis comparisons. Of the 17 KEGG level-2 and 116 of level-3 pathways that differed between contaminated and both uncontaminated areas according to the Kruskal-Wallis tests, all were found to be associated with the CH (14 pathways from level-2 and 80 level-3) or CL/KL (3 level-2 and 36 level-3) areas by a PLS analysis (Figure 2c, Supplementary Table 11).

**Supplementary References**

Barter R. (2017). A graphical tool for exploring complex datasets using heatmaps. https://cran.r-project.org/web/packages/superheat/superheat.pdf.

Barton K. (2016). MuMIn: Multi-model inference. R Package Version 1.15.6. https://cran.r-project.org/web/packages/MuMIn/MuMIn.pdf. https://cran.r-project.org/web/packages/MuMIn/MuMIn.pdf.

Benjamini Y, Hochberg Y. (1995). Controlling the false discovery rate: a practical and powerful approach to multiple testing. *J R Stat Soc Ser B* **57**: 289–300.

Bokulich NA, Subramanian S, Faith JJ, Gevers D, Gordon JI, Knight R, *et al.* (2013). Quality-filtering vastly improves diversity estimates from Illumina amplicon sequencing. *Nat Methods* **10**: 57–9.

Bolker BM, Brooks ME, Clark CJ, Geange SW, Poulsen JR, Stevens MHH, *et al.* (2009). Generalized linear mixed models: a practical guide for ecology and evolution. *Trends Ecol Evol* **24**: 127–135.

Caporaso JG, Kuczynski J, Stombaugh J, Bittinger K, Bushman FD, Costello EK, *et al.* (2010). QIIME allows analysis of high-throughput community sequencing data. *Nat Methods* **7**: 335–336.

Caporaso JG, Lauber CL, Walters WA, Berg-Lyons D, Huntley J, Fierer N, *et al.* (2012). Ultra-high-throughput microbial community analysis on the Illumina HiSeq and MiSeq platforms. *ISME J* **6**: 1621–1624.

Chesser RK, Bondarkov M, Baker RJ, Wickliffe JK, Rodgers BE. (2004). Reconstruction of radioactive plume characteristics along Chernobyl’s Western Trace. *J Environ Radioact* **71**: 147–157.

Dinno A. (2016). dunn.test: Dunn’s test of multiple comparisons using rank sums. R package version 1.3.3. https://CRAN.R-project.org/package=dunn.test. https://cran.r-project.org/package=dunn.test.

Edgar RC, Haas BJ, Clemente JC, Quince C, Knight R. (2011). UCHIME improves sensitivity and speed of chimera detection. *Bioinformatics* **27**: 2194–2200.

Kallio ER, Begon M, Birtles RJ, Bown KJ, Koskela E, Mappes T, *et al.* (2014). First report of *Anaplasma phagocytophilum* and *Babesia microti* in rodents in Finland. *Vector Borne Zoonotic Dis* **14**: 389–93.

Koropatkin NM, Cameron EA, Martens EC. (2012). How glycan metabolism shapes the human gut microbiota. *Nat Rev Microbiol* **10**: 323.

Kozakiewicz M, Chołuj A, Kozakiewicz A. (2007). Long-distance movements of individuals in a free-living bank vole population: an important element of male breeding strategy. *Acta Theriol (Warsz)* **52**: 339–348.

Langille M, Zaneveld J, Caporaso JG, McDonald D, Knights D, Reyes J, *et al.* (2013). Predictive functional profiling of microbial communities using 16S rRNA marker gene sequences. *Nat Biotechnol* **31**: 814–21.

Lehmann P, Boratyński Z, Mappes T, Mousseau TA, Møller AP. (2016). Fitness costs of increased cataract frequency and cumulative radiation dose in natural mammalian populations from Chernobyl. *Sci Rep* **6**: 19974.

Lozupone C, Lladser ME, Knights D, Stombaugh J, Knight R. (2011). UniFrac: an effective distance metric for microbial community comparison. *ISME J* **5**: 169–172.

Maurice CF, Cl Knowles S, Ladau J, Pollard KS, Fenton A, Pedersen AB, *et al.* (2015). Marked seasonal variation in the wild mouse gut microbiota. *ISME J* 1–12.

McDonald D, Price MN, Goodrich J, Nawrocki EP, DeSantis TZ, Probst A, *et al.* (2012). An improved Greengenes taxonomy with explicit ranks for ecological and evolutionary analyses of bacteria and archaea. *ISME J* **6**: 610–618.

McMurdie PJ, Holmes S. (2013). Phyloseq: An R package for reproducible interactive analysis and graphics of microbiome census data. *PLoS One* **8**. e-pub ahead of print, doi: 10.1371/journal.pone.0061217.

Morton JT, Sanders J, Quinn RA, McDonald D, Gonzalez A, Vázquez-Baeza Y, *et al.* (2017). Balance Trees reveal microbial niche differentiation. *mSystems* **2**. http://msystems.asm.org/content/2/1/e00162-16 (Accessed August 9, 2017).

Møller AP, Mousseau TA. (2013). Assessing effects of radiation on abundance of mammals and predator–prey interactions in Chernobyl using tracks in the snow. *Ecol Indic* **26**: 112–116.

Oksanen J, Blanchet F, Kindt R, Legendre P, O’Hara R. (2016). Vegan: community ecology package. *R Packag 23-3* Available at: https://cran.r-project.org/web/packa.

Weiss S, Xu ZZ, Peddada S, Amir A, Bittinger K, Gonzalez A, *et al.* (2017). Normalization and microbial differential abundance strategies depend upon data characteristics. *Microbiome* **5**: 27.

Zeng B, Han S, Wang P, Wen B, Jian W, Guo W, *et al.* (2015). The bacterial communities associated with fecal types and body weight of rex rabbits. *Sci Rep* **5**: 9342.

Zhang J, Kobert K, Flouri T, Stamatakis A. (2014). PEAR: A fast and accurate Illumina Paired-End reAd mergeR. *Bioinformatics* **30**: 614–620.

**Supplementary Figures**

**Supplementary Figure 1**

**
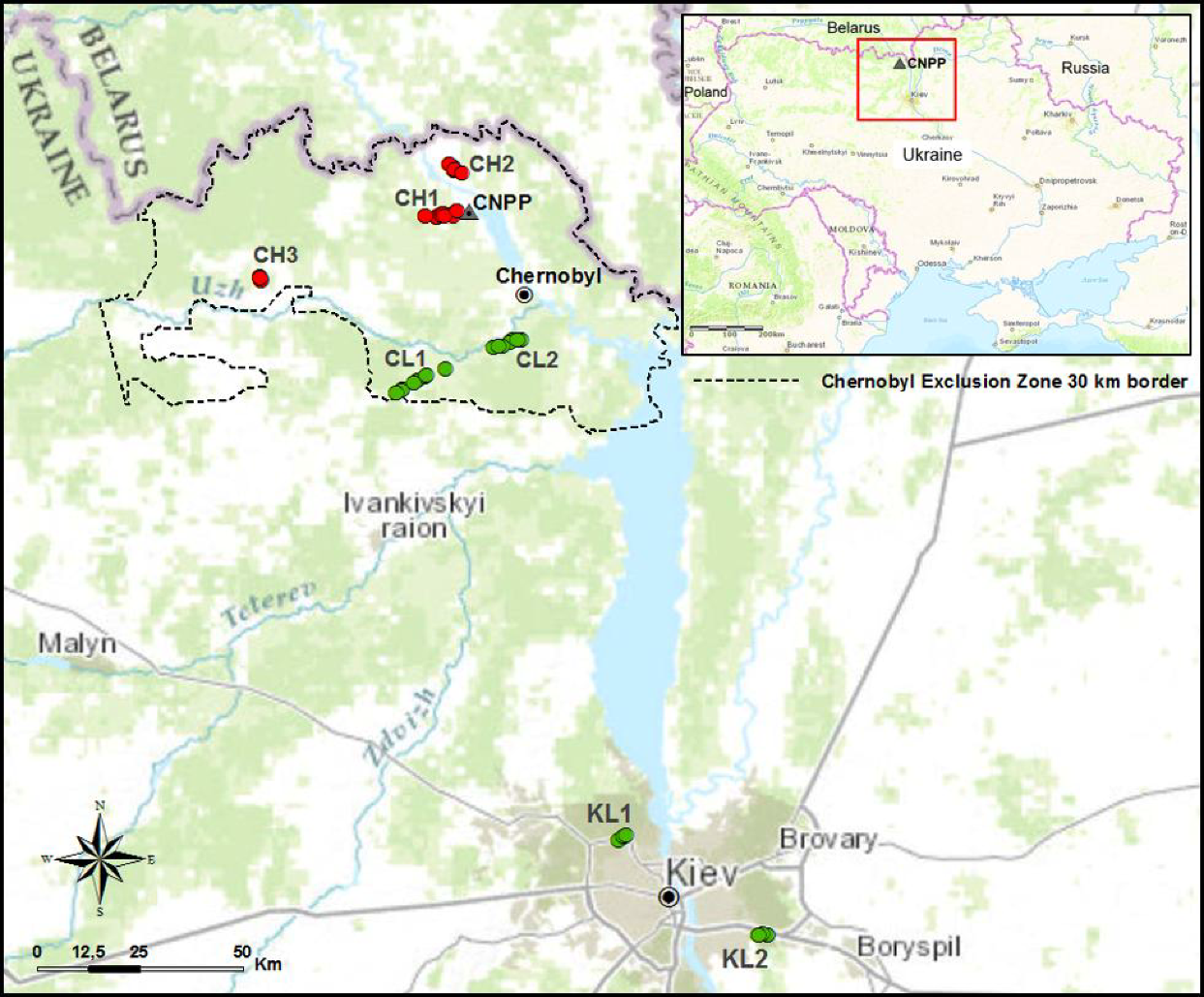
**

**Supplementary Figure 1.** Map of the study areas with bank vole trapping locations shown by points (n=62). Replicate sites within each area (*e.g.* CH1-3, CL1-2 and KL1-2) are shown, with areas contaminated (CH) and uncontaminated (CL) with radionuclides within the Chernobyl Exclusion Zone and uncontaminated area near Kyiv (KL), Ukraine. Color of the point indicates differences in environmental radiation levels, CH, red (10–198.7 µSv/h); CL, green (0.1–0.6 µSv/h) and KL, green (0.2–0.6 µSv/h). Supplementary Figure 1 was created using ESRI ArcGIS (v10.5).

**Supplementary Figure 2**

**
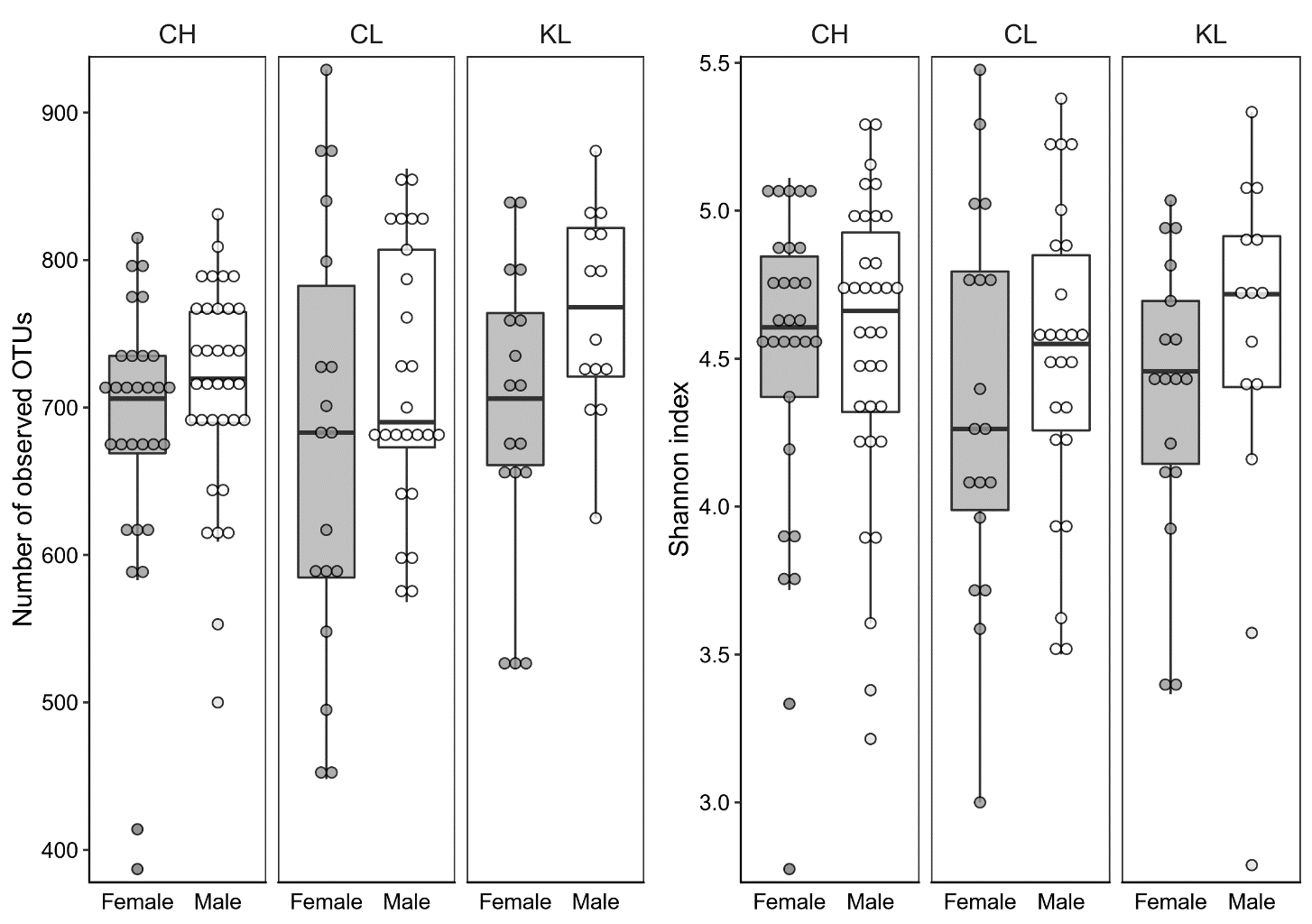
**

**Supplementary Figure 2.** Measures of alpha diversity for the gut microbiota of bank vole females and males inhabiting areas that differ in levels of environmental radiation. Box-and-whisker plots represent the median and interquartile range of alpha diversity estimates (*i.e.* number of observed OTUs, Shannon index). Each point represents a single sample from contaminated (CH) and uncontaminated (CL) with radionuclides areas within the Chernobyl Exclusion Zone and uncontaminated area near Kyiv (KL), Ukraine (Bonferroni-corrected Kruskal–Wallis test). All comparisons were non-significant.

**Supplementary Figure 3**

**
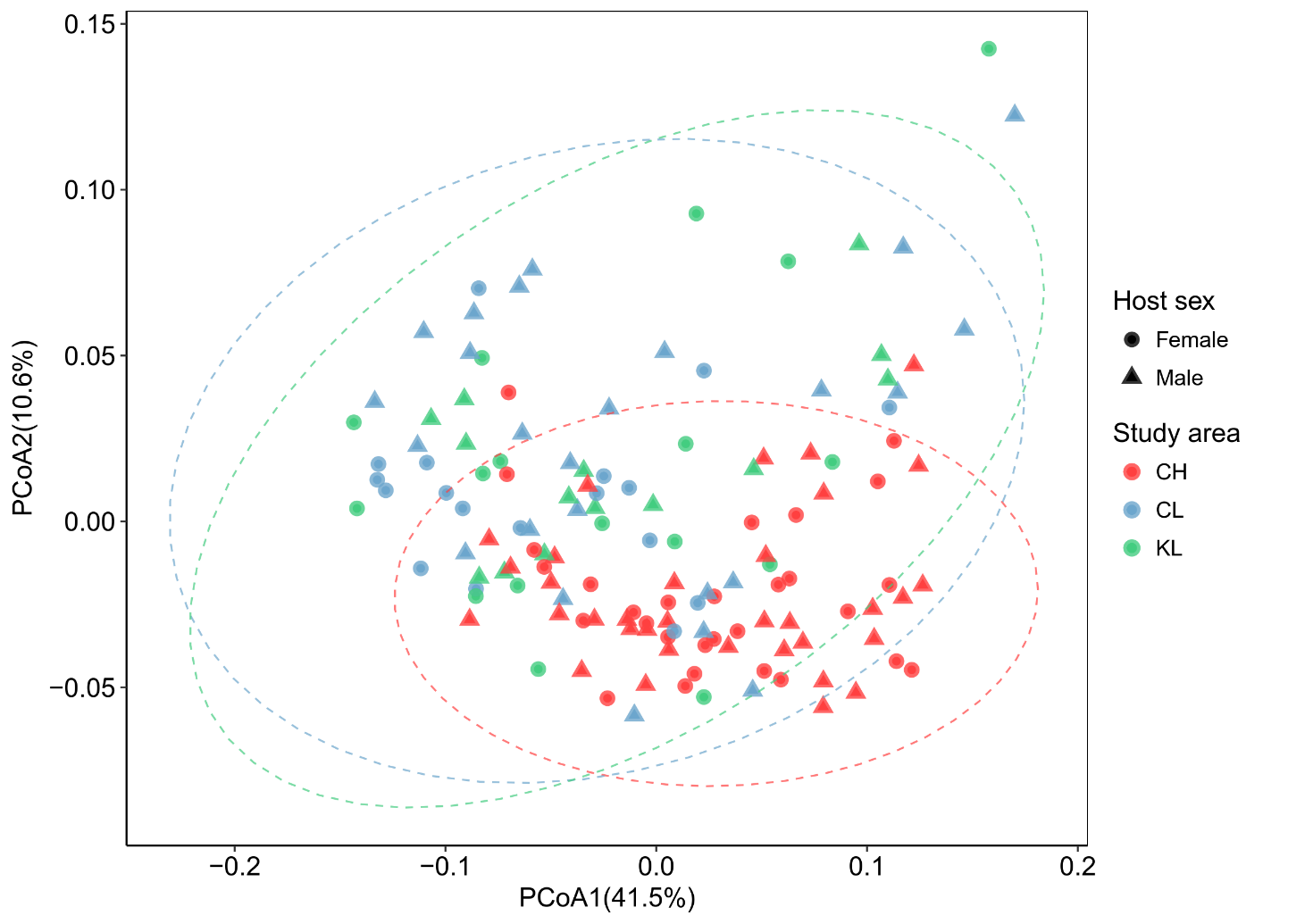
**

**Supplementary Figure 3.** Differences in bank vole gut microbiota beta diversity associated with environmental radiation exposure. PCoA on weighted UniFrac distances between bank vole gut microbiota profiles among the three study areas that differ in levels of environmental radioactivity are shown along the first two PC axes. Each point represents a single sample, shape indicate host sex, colored according to study area: CH, red (n=63); CL, blue (n=43); KL, green (n=31). Ellipses represent a 95% CI around the cluster centroid. Clustering significance by treatment group was determined by adonis; *P*<0.001.

**Supplementary Figure 4**

**
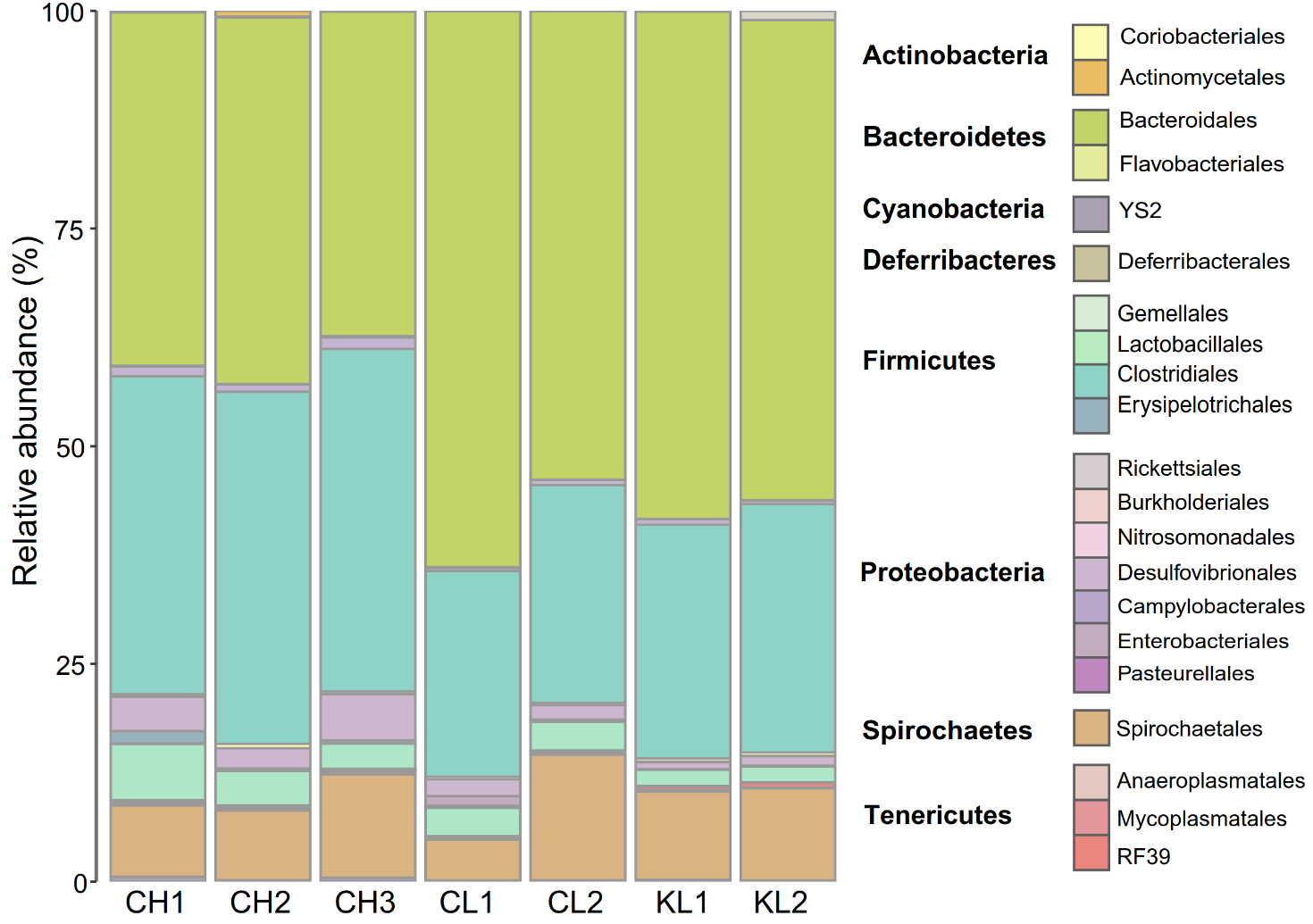
**

**Supplementary Figure 4.** Mean relative abundance of bacterial taxa at order level from bank voles inhabiting areas that differ in levels of environmental radiation. Shown are the replicate sites within each study area contaminated (CH1-3) and uncontaminated (CL1-2) with radionuclides within the Chernobyl Exclusion Zone and an uncontaminated area near Kyiv (KL1-2), Ukraine. Taxa are identified in the legend. Unassigned taxa (<2.32%) are not shown.

**Supplementary Figure 5**

**
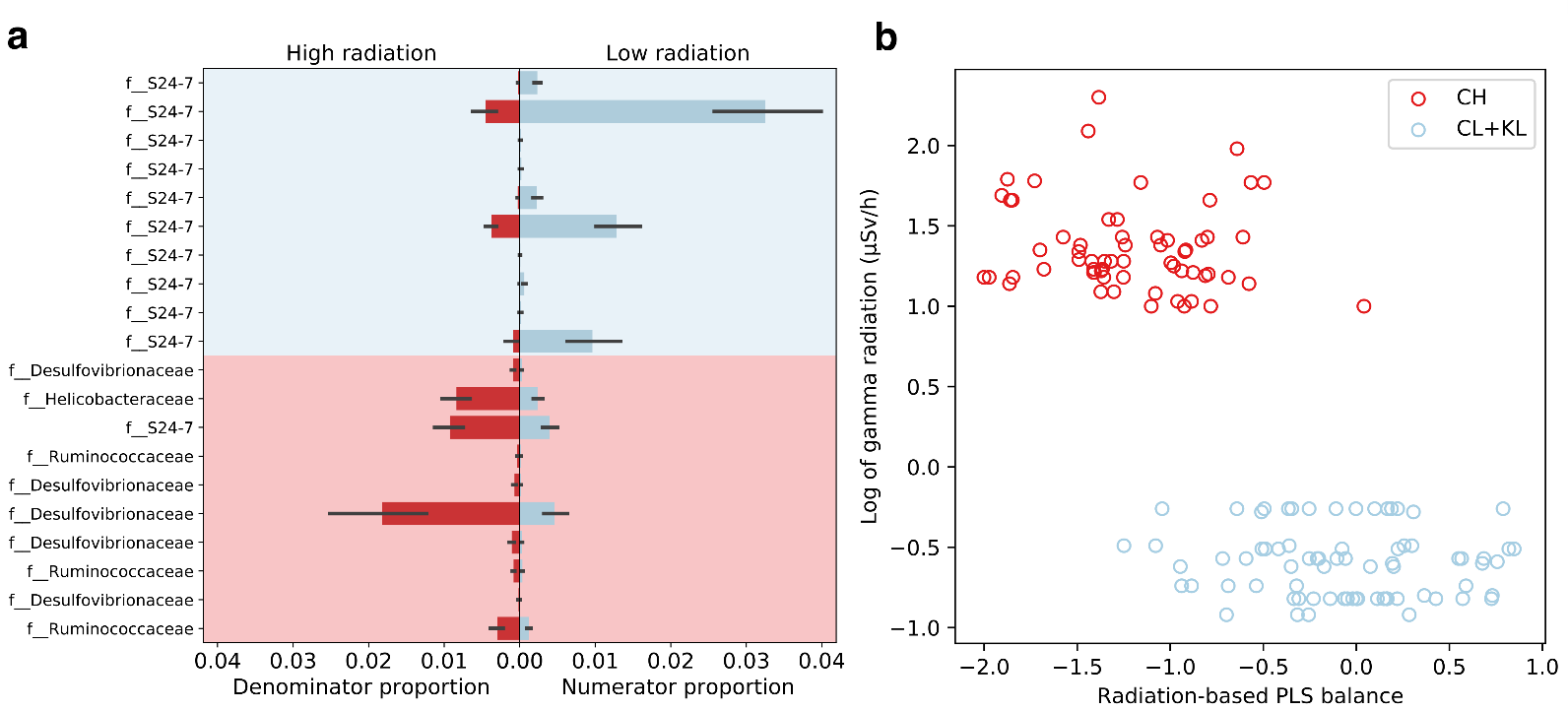
**

**Supplementary Figure 5.** Partial least squares (PLS) balance analysis of radiation-differentiated OTUs, and classification of samples to radiation levels based on OTU composition. (a) The radiation-based PLS balance comprised 281 OTUs associated with low radiation (log radiation<0), with a high representation of taxa from family *S24-7* and 175 OTUs associated with high radiation (log radiation>0) with a high representation of taxa from the order Clostridiales; shown are the proportions in the two groups of the top 10 OTUs in each group based on PLS score. (b) The radiation-based balance was negatively correlated with radiation (Pearson’s *r* =–0.766, *P*-value=1.46x10^–27^) with a mean Q^2^ score of 0.439.

**Supplementary Figure 6**

**
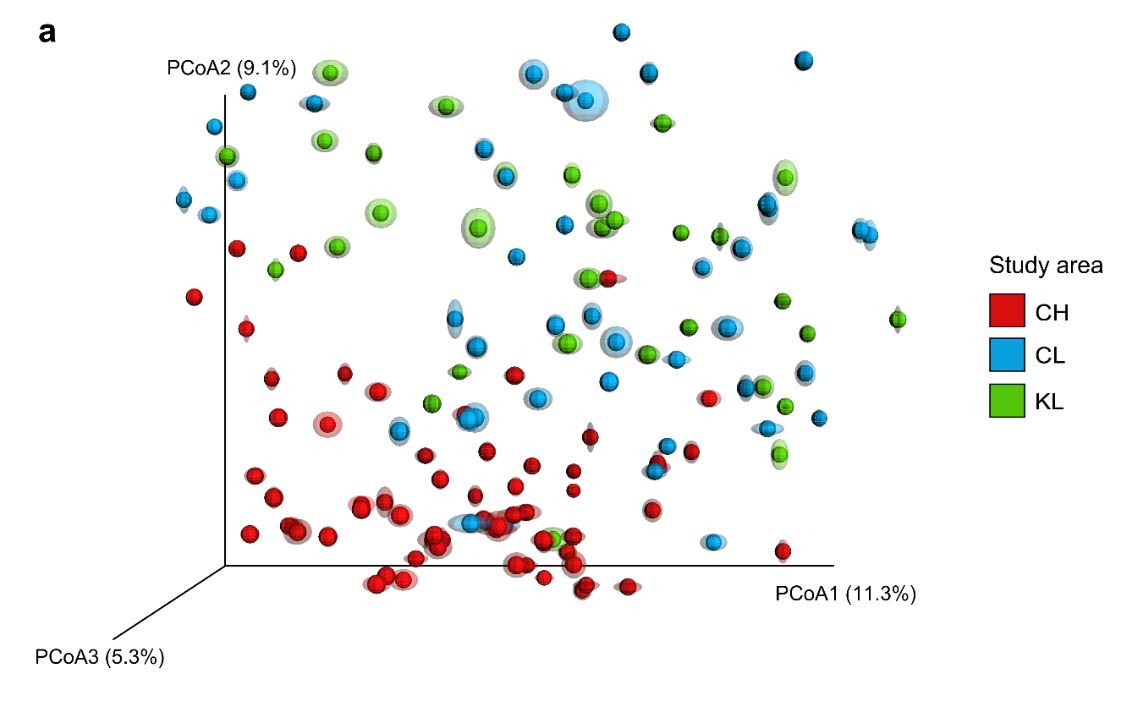

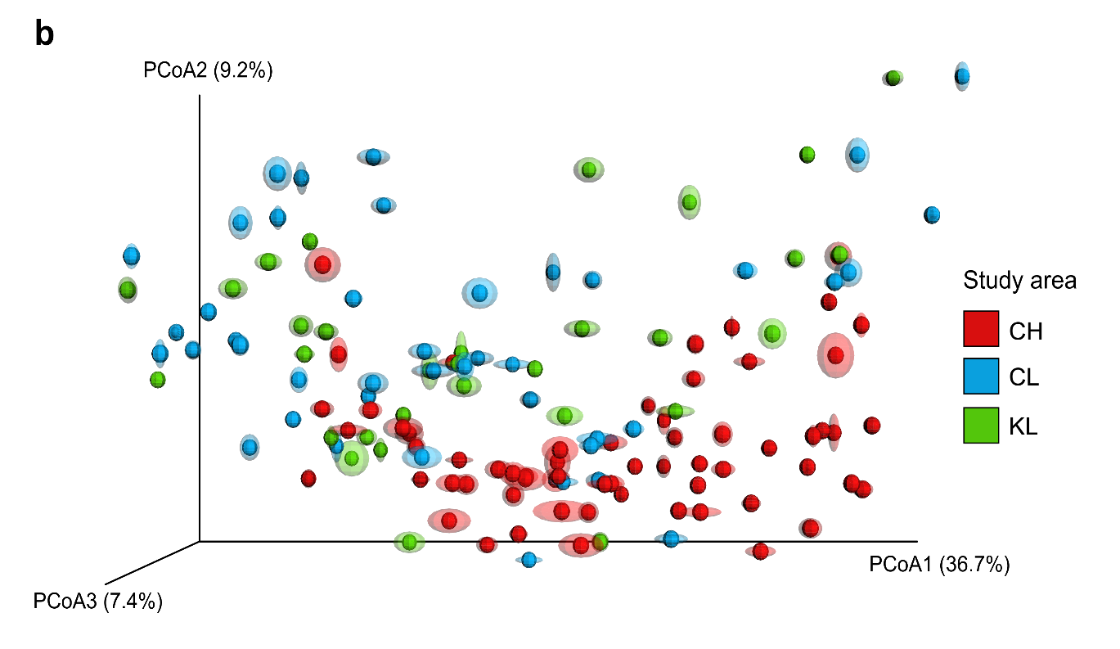
**

**Supplementary Figure 6.** Differences in bank vole gut microbiota beta diversity associated with environmental radiation exposure. Jackknifed PCoA plot of (a) Bray-Curtis and (b) weighted UniFrac distances between sample groups. Jackknife resampling was performed 10 times at a depth of 1800 reads per sample. Each point represents the microbiome from an individual bank vole. Semi-transparent areas represent confidence ellipses (inter-quartile range) generated by jackknifing.

**Supplementary Tables**

**Supplementary Table 1.** 16S rRNA gene sequencing metadata.

**Supplementary Table 2.** Statistical comparison of alpha diversity estimates by Kruskal–Wallis tests using Dunn’s *post hoc* test and followed by a Bonferroni correction for the significance level.

**Supplementary Table 3.** Permutational MANOVA (PERMANOVA) statistical tests on weighted UniFrac (wUniFrac) distances and Bray-Curtis dissimilarity for gut microbial communities of bank voles inhabiting areas that differ in environmental radiation levels.

**Supplementary Table 4.** Final GLMs that show significant predictors of abundance of bacterial phylum within the gut microbiota of bank voles inhabiting the area surrounding the former Chernobyl nuclear power plant and areas near Kyiv, Ukraine. Only significant models are shown, with significant *P*-values shown in bold. AIC fit criterion is given for each full model below.

**Supplementary Table 5.** Relative abundance (average of taxonomic groups abundance within each sample) of Phyla, Classes, Orders, Families, Genera for wild-caught bank voles (*Myodes glareolus*) from contaminated (CH) and uncontaminated (CL) with radionuclides areas within the Chernobyl Exclusion Zone and uncontaminated area near Kyiv (KL), Ukraine.

**Supplementary Table 6.** Summary of gut microbiota of the Phyla, Classes, Orders, Families and Genera for wild-caught bank voles (*Myodes glareolus*), with significantly different relative abundances (Kruskal-Wallis, Bonferroni-adjusted *P*<0.05) among the study areas (e.g. CL, KL and CH).

**Supplementary Table 7.** List of OTUs in the numerator (CL/KL) and denominator (CH) of the area-based PLS balance, including PLS scores and taxonomic assignments for each OTU.

**Supplementary Table 8.** Summary statistics for area-based PLS balance: area under the curve (AUC), mean area under the curve across all cross validation runs. *F*-statistic, *P*-value quantifying sample separation from an ANOVA test. AUC scores for each cross-validation run are also included. In addition, summary statistics for the random sampling (5 random subsamples of n=30 samples per study area and 5 random subsamples of n=10 samples per study area) of the bank vole gut microbiome samples (with replacement) can be found in the random sampling sheet.

**Supplementary Table 9.** List of OTUs in the numerator (low radiation) and denominator (high radiation) of the radiation-based PLS balance, including PLS scores and taxonomic assignments for each OTU.

**Supplementary Table 10.** Summary statistics for radiation-based PLS balance: Pearson’s r, *P*-value and Q2_CV to quantify the mean predicted sum of squares for each cross validation run. Q2 scores for each cross-validation run are also included.

**Supplementary Table 11.** Statistical comparison of functional predictions by PICRUSt grouped at the level-2 and level-3 of KEGG pathways using Kruskal–Wallis tests and Dunn’s *post hoc* test and followed by a Benjamini-Hochberg False Discovery Rate (FDR) correction for the significance level and the PLS analysis which classify inferred KEGG pathways to the study area (*i.e.* CH vs CL/KL).
